# The evolution of eyes and visually guided behaviour

**DOI:** 10.1098/rstb.2009.0083

**Published:** 2009-10-12

**Authors:** Dan-Eric Nilsson

**Affiliations:** Department of Cell and Organism Biology, Lund University, 22362 Lund, Sweden

**Keywords:** vision, evolution, eye, visual task, visual information, photoreceptor

## Abstract

The morphology and molecular mechanisms of animal photoreceptor cells and eyes reveal a complex pattern of duplications and co-option of genetic modules, leading to a number of different light-sensitive systems that share many components, in which clear-cut homologies are rare. On the basis of molecular and morphological findings, I discuss the functional requirements for vision and how these have constrained the evolution of eyes. The fact that natural selection on eyes acts through the consequences of visually guided behaviour leads to a concept of task-punctuated evolution, where sensory systems evolve by a sequential acquisition of sensory tasks. I identify four key innovations that, one after the other, paved the way for the evolution of efficient eyes. These innovations are (i) efficient photopigments, (ii) directionality through screening pigment, (iii) photoreceptor membrane folding, and (iv) focusing optics. A corresponding evolutionary sequence is suggested, starting at non-directional monitoring of ambient luminance and leading to comparisons of luminances within a scene, first by a scanning mode and later by parallel spatial channels in imaging eyes.

## 1. Background

### (a) Introduction

New molecular and genetic data on eye evolution are being generated at a continuously increasing pace. This has led to a much improved understanding of eye evolution, but also to frequent conflicts between data of different types or from different species. In particular, morphological data are sometimes pointing in one direction and molecular cues in another. But both types of data are, in principle, equally valid and true. The reason that different correct data sometimes appear to be in conflict with each other is of course that our interpretations are incorrect, or at least not complete. In this paper, I briefly review the information most relevant to early eye evolution and discuss possible scenarios in the light of functional arguments.

Without an understanding of how selection has guided the process of evolution, any evolutionary scenario is incomplete. Gene or protein phylogenies can tell us what is likely to have happened at the molecular level, and other approaches such as expression studies and knockout experiments can relate these molecular changes to functions. But to understand why features or functions have evolved, we need plausible ideas about their selection, so that we can explain why the presumed evolutionary paths were favoured at the expense of other possibilities. In the evolution of sensory functions, reception of external information will only be favoured if it is used for controlling the behaviour or the physiology such that it has a positive effect on fitness.

To analyse the benefit of sensory systems, it is useful to introduce the term ‘sensory task’, broadly defined as a systematic behavioural or physiological response to specific information received. The simplest possible sense involves a single sensory task, linking specific received information to a single specific response. In the evolution of a sensory system, a single sensory task is also the obvious starting point. Apart from later modifications of the sensory task, subsequent evolutionary elaboration of the sensory system will be associated with the appearance of new tasks. In this way, sensory evolution can be understood as a sequential addition of sensory tasks. The basic evolutionary principle of duplication followed by modification would be a typical mechanism for adding new tasks to a sensory system.

Sensory tasks can be based on simple receptor–effector circuits without intermediate processing, or they can involve comparisons or memory, requiring various degrees of intermediate processing from simple to exceedingly complex. In a reconstruction of sensory evolution, it is natural to assume that the simple and less demanding tasks evolved first, because they require the smallest number of components. As the sensory tasks accumulate, the sensory organs will have to change from delivering specific information, tuned for one or a few similar tasks, to providing more general information, covering the requirements of a broad range of different tasks. This process will increase the demands on the nervous system, because when the received information gradually becomes more general, the need increases for sorting and filtering the information to suit each individual task.

Each sensory task requires that a specific stimulus be connected to a specific response. For this to work, it is necessary that the sensory system is built such that it responds reliably when the specific stimulus is present, but ignores stimuli that do not fit the specific requirements. The sensory system should thus act as a filter that passes the specific stimulus, but blocks all other information. This concept of sensory filtering was developed by Wehner (1987), who also coined the term ‘matched filters’ to collectively include the sensory and neural properties that together form a filter, which matches the specific information pattern that elicits a behavioural response. Each sensory task will thus require a specific matched filter, and in simple sensory systems, supporting only one or a few similar sensory tasks, these filters can be implemented in the properties of the receptor cells or the design of the sensory organ (for example, in vision, see Nilsson *et al*. 2005; Land & Nilsson 2006). When a sensory organ evolves to support a large number of sensory tasks, the matched filters will gradually have to move from the sensory organ to the nervous system. In vision, this means that evolution can be expected to proceed from single-purpose photoreceptive systems with peripheral filtering to general-purpose eyes with central neural filtering (Land & Nilsson 2006).

Sensory systems can improve fitness only through the responses they trigger. Conversely, behaviours and physiological responses require sensory control to be elicited specifically under conditions where the response improves the situation. Thus, sensors and effectors make sense only in combination, and evolution of the senses is intimately linked to the evolution of locomotion and behaviour. The ability to acquire, process and respond differentially to large amounts of information is also what sets animals apart from all other forms of life, and the story of animal evolution is largely a story of sensory evolution. In fact, most ecosystems would have been very different without animals and their senses. Therefore, every attempt to understand sensory evolution is also an attempt to understand the evolution of animals and the changing ecosystems they have been parts of.

In this paper, I will focus on the early stages of photoreceptor evolution that led up to the first eyes. Existing knowledge and theories from morphology, physiology and molecular biology will form the base, and I will investigate how this can be combined with the constraints and possibilities of a sequential evolution of visual tasks. Some of the questions that will be probed are: which were the original sensory tasks that led to evolution of the first eyes; in which sequence did these sensory tasks evolve and what did these tasks require in terms of sensory structures and functions. The aim of the paper is to extend the discussion of early eye evolution from ‘how’ to ‘why’ questions.

### (b) Molecular and morphological cues to eye evolution

Theories of how eyes originated and evolved must of course be based on the known diversity of present day visual systems and other light-sensitive systems in animals. Here, I will not attempt a detailed review of this enormous diversity, but some of the more crucial aspects will be briefly mentioned (for recent reviews, see Arendt & Wittbrodt 2001; Arendt 2003; Plachetzki *et al*. 2005; Gregory 2008). The most fundamental and presumably most ancient property of visual systems is the ability to sense light. Across all organisms, there is a handful of different types of organic molecules that are involved in sensing light (Björn 2007), and they are typically bound to devoted proteins. With the possible exception of light sensing by cytochrome c oxidase in sponge larvae (Björn & Rasmusson 2009), animal light sensing is based on either cryptochromes or opsins. Whereas non-visual photoreception in animals may use either cryptochromes or opsins, vision is exclusively based on opsin photopigments. Animal opsins (type-II opsins) are unique to animals and apparently unrelated to bacterial and algal opsins (type-I opsins; Larusso *et al*. 2008).

Animal opsins belong to the G-protein-coupled receptor (GPCR) family, where the great majority of proteins bind and respond to chemical ligands, and are involved in synaptic transmission, hormone reception, olfaction or taste (Pierce *et al*. 2002). The difference between GPCRs detecting chemical stimuli and the light-sensitive opsins is that the opsins bind a light-sensitive chromophore and signal when the chromophore has been altered by light. In opsins, the chromophore is a vitamin-A derivative, most commonly vitamin-A aldehyde, also known as retinal, which responds to light by changing from an 11-*cis* to an all-*trans* conformation. Opsins are thus still behaving as chemoreceptor proteins, but for a specific light-induced chemical stimulus, and it is possible that this reflects a chemoreceptor origin of animal opsins.

There are different classes of opsins that behave in different ways when the chromophore is isomerized. The c-opsins (originally known from vertebrate rods and cones) release the chromophore after it has been converted to the all-*trans* isoform (Lamb & Pugh 2004), whereas the r-opsins keep both the 11-*cis* and the all-*trans* isoforms firmly bound (r-opsins are known from the photoreceptors of invertebrate eyes and also represented by melanopsins in vertebrates). In the r-opsins, the chromophore can be converted back to the 11-*cis* form by the absorption of yet another photon, and this photoregeneration serves to replenish sensitive photopigment (Hamdorf 1977). The c-opsins cannot themselves regenerate the chromophore, and a separate enzymatic system is required for this purpose (Lamb & Pugh 2004). A functionally aberrant class of opsins acts not as receptor proteins but as photoisomerases that use light to convert chromophore from the all-*trans* to the 11-*cis* form, which is then released and ready to be incorporated in a conventional opsin (Sperling & Hubbard 1975; Smith & Goldsmith 1991; McBee *et al*. 2001; Gonzalez-Fernandez 2003).

Opsins from different classes also couple to distinct G-proteins, which are associated with different transduction cascades (table 1). Bilaterians are believed to generally possess at least three opsin classes—c-opsins, r-opsins and G_o_-opsins/photoisomerases (Terakita 2005; Raible *et al*. 2006)—whereas cnidarians have c-opsins and a unique class, the cnidops class, that seems to be the cnidarian counterpart of both r-opsins and G_o_-opsins/photoisomerases (Plachetzki *et al*. 2007; Koyanagi *et al*. 2008; Kozmik *et al*. 2008; Suga *et al*. 2008). Because opsin genes are absent from sponges, placozoans and choanoflagellates, but sponges have other GPCRs (Suga *et al*. 1999; Plachetzki *et al*. 2007), it seems that opsins originated in early eumetazoans and that the different classes radiated partly before and partly after the split between Cnidaria and Bilateria.

**Table 1.** Opsins and transduction cascades in animal photoreceptors. PDE, phosphodiesterase; PLC, phospholipase C; GC, guanylate cyclase; AC; adenylate cyclase; cGMP, cyclic guanosine phosphate; cAMP, cyclic adenosine phosphate; IP_3_, inositol triphosphate; DAG, diacylglycerol. For more information, see Pierce *et al*. (2002) and Terakita (2005).

opsin	G-protein	controlled enzyme	second messenger	response polarity
c-opsin	G_t_	PDE	cGMP	hyperpolarization
r-opsin	G_q_	PLC	IP_3_, DAG	depolarization
G_o_-opsin	G_o_	GC	cGMP	hyperpolarization/depolarization
cnidops class	G_s_	AC	cAMP	depolarization

Another common feature of animal eyes is that they share the use of homologous developmental genes, in particular, the *Pax-6* gene (Quiring *et al*. 1994; Halder *et al*. 1995; Gehring & Ikeo 1999). This has been taken as evidence that all eyes are strictly homologous organs (Gehring & Ikeo 1999; Gehring 2005), but because *Pax-6* and other developmental genes are activated repeatedly during the ontogenetic development, it is not clear whether the original role of these genes was to specify a sensory/nervous system, a photoreceptor cell or an eye (Nilsson 1996, 2004; Fernald 2000, 2004; Kozmik 2005). The developmental genes, therefore, are less informative about eye evolution than are the opsins.

Almost without exception, the photoreceptor cells of animal eyes have conspicuous membrane specializations for housing large quantities of opsin photopigment, which is membrane bound. The two most common types of such membrane specializations are those based on modified cilia and those based on microvilli projecting from the cell body. The resulting distinction between ciliary and rhabdomeric photoreceptor cells has had a great impact on our understanding of eye evolution (Land & Fernald 1992; Arendt & Wittbrodt 2001; Nilsson 2004). Most invertebrate eyes are based on rhabdomeric receptor cells, whereas vertebrate and cnidarian eyes are based on ciliary receptor cells. The distinction between ciliary and rhabdomeric receptor cells is emphasized by the fact that they strictly employ different classes of opsin, c-opsin and r-opsin, respectively, and as a consequence, they also use different transduction cascades (table 1). It is now clear that bilaterian animals typically have both types of photoreceptor cells, but it depends on the phylum as to which type is used for vision (Arendt 2003; Arendt *et al*. 2004; Plachetzki *et al*. 2005). The situation is complicated by the existence of more than two types of receptor cells, by intermediate types and by cells that change from one type to another during ontogenetic development (Eakin & Westfall 1965; Eakin 1972; Blumer 1995, 1996). A reasonable conclusion is thus that different types of photoreceptor cells were established early in the eumetazoan evolution, but that later modification in cell structure, molecular mechanisms and use, together with genetic co-option, has blurred the picture to varying degrees in different corners of the animal kingdom.

**Figure 1.**
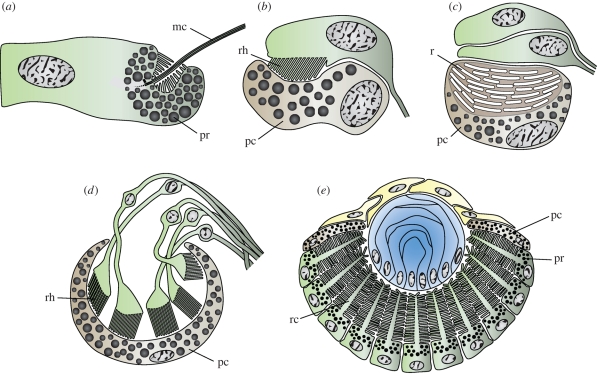
Semi-schematic drawings of ocelli and simple eyes: (*a*) single cell ocellus of box jellyfish larva; (*b*) polychaete larval ocellus; (*c*) ocellus of acoel flatworm, with reflecting platelets in the pigment cell, but no membrane stacking in the two receptor cells; (*d*) inverse cup eye of planarian flatworm and (*e*) everse lens eye of juvenile box jellyfish. Photoreceptor cells are indicated by green shading. pr, screening pigment in receptor cell; mc, motile cilium; pc, specialized pigment cell; rh, rhabdom; r, reflective crystals; rc, receptive cilium.

Earlier distinctions between eyes with inverse or everse retinas, defined by the orientation of the receptor cells (Salvini-Plawen & Mayr 1977; Arendt & Wittbrodt 2001), also suffer from cases that are intermediate or otherwise hard to interpret, and presently, it seems that the structure and design of eyes are so different between animal phyla that, for the most part, eye evolution has proceeded independently in different animal groups. For an understanding of the early phases of eye evolution, it is of more interest to consider molecularly specified types of photoreceptor cells (Arendt 2003, 2008) and their position and roles in different extant animals. In this paper, I make the distinction between non-visual photoreception and vision. The former includes non-neural cells that express opsins for their physiological control, but also photoreceptor neurons that are not incorporated into eyes, and not associated with screening pigment or other screening structures. Such cells will not gain any information about the direction of light, but will respond to the general ambient intensity. A prerequisite for visual photoreception is that it provides information about the direction of light, either by partially shielded photoreceptors and scanning body movements (figure 1*a*–*c*) or by comparison of signals from multiple photoreceptors in eyes (true spatial vision; figure 1*d*,*e*).

### (c) Types of processes in eye evolution

**Figure 2.**
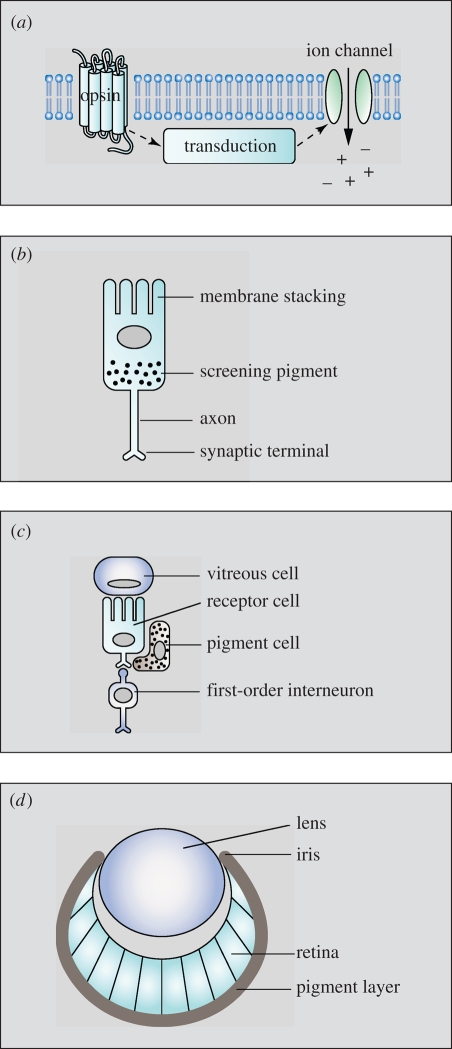
The four processes (*a*, molecular components; *b*, cell structures; *c*, cell types; *d*, organ shape) involved in eye evolution. The processes overlap in time, but are initiated in the ascending order.

Eyes, just as other complex organs, are composed of different specialized cells that have cell morphologies and biochemical mechanisms needed for their specific function. Obviously, some structures and molecular mechanisms need to be in place before it makes sense for others to evolve. In terms of eye evolution, it is clear that light sensitivity must have evolved to some degree before receptor-cell structures start to evolve, and receptor cells must, in turn, exist before a multi-cellular eye can be assembled. In this sequence of events, four different evolutionary processes come into play: (i) evolution of molecular components, (ii) evolution of cell structures, (iii) evolution of cell types, and (iv) evolution of organ shape (denoted *a*–*d* in figure 2). The four processes overlap and interact, but each process introduces change in its own particular way.

The molecular components such as opsins and the transduction proteins are subject to variations in the protein sequence, introduced by random mutations. Selection acts on sequence changes, and because the vast majority of changes are neutral or detrimental, the small choice available to selection will make the direction of sequence evolution depend largely on the random nature of mutations, and the process generates discrete rather than continuous change. Genes may have more than one role, and this gene sharing (Piatigorsky 2007) has important consequences for change. Classical examples of gene sharing in vision are stress proteins or chaperones that also function as crystallins in the lens or cornea (Piatigorsky 1998). The more extensive the gene sharing is, the larger is the chance that a sequence change has some positive effects, but the risk of negative consequences is likewise increased (Piatigorsky 2007). The remedy for this Gordian knot is gene duplication, in which different copies are free to specialize for different roles. Gene duplication without previous gene sharing is of course also possible, but a less powerful evolutionary mechanism. Gene duplication and subsequent modification are responsible for the many different types of opsins (Terakita 2005).

Evolution of cell structures such as rhabdoms, modified cilia or axons is necessary for particular functions or at least for improving the efficiency of functions. Changes in developmental genes are ultimately responsible for the coordinated expression of proteins that form the cell structures. The gradual and quantitative nature of such modifications is typically the result of interactions between many genes (Futuyma 1986), and as such, the randomness of mutations would be expected to have only minor impact on the evolution of these features.

Evolution of cell types, which can most often be described as cell specialization, generally requires a growing number of separate cell types. Cell-type duplication followed by segregation is thought to be a central mechanism in the evolution of metazoan complexity (Arendt 2008; Arendt *et al.* 2009). The genetic basis is subspecification of cell types, through modifications of patterning genes, followed by a more specialized expression profile of each new cell type. It is believed that many of the specialized cells in animal retinas have originated from ancestral cells with multiple functions, much like changing from a one-man band to a large orchestra. Such principles are believed to be responsible for the evolution of different receptor cell types, pigment cells and interneurons from ancestral receptor cells (Plachetzki & Oakley 2007; Arendt 2008; Erclik *et al*. 2008). A somewhat different mode of cell-type divergence is evident in the retinas of many animals and results in a gradual change in cell morphology from one part of the retina to another. A clear example is found in the lens eyes of box jellyfish, where all of the ciliary receptor cells contain dark screening pigment. Towards the periphery of the retina, the ciliary segments become smaller, and there is a gradual transition from pigmented receptor cells to the pure pigment cells, totally lacking cilia, that form the iris of the eye (O'Connor *et al*. 2009).

The shape and size of an eye are of crucial importance for its optical performance. Organ design parameters are controlled by the proliferation and growth of cells, and in analogy with the evolution of complex cell structures, many different genes are likely to be involved in organ morphology. This makes the variation within the population virtually continuous (Futuyma 1986). Selection from a continuous (not discrete) variation is the reason why eyes can be so precisely tuned to perform close to the physical limits (Nilsson & Pelger 1994; Land & Nilsson 2002).

Plachetzki & Oakley (2007) emphasize ‘tree thinking’, with duplication and divergence, as a complement to traditional linear models of eye evolution. As should be obvious from the reasoning mentioned earlier, both conceptual frameworks are necessary for understanding eye evolution. Processes (*a*) and (*c*) of figure 2 follow the concept of tree thinking, whereas processes (*b*) and (*d*) follow a linear model of evolution. As I will argue in the next section, the evolution of sensory systems is always a punctuated process, in which behavioural tasks define the entities on which selection ultimately works.

Much of the eye evolution literature is concerned with questions of homology (Arendt 2003, 2008; Gehring 2005; Plachetzki *et al*. 2005; Plachetzki & Oakley 2007; Gregory 2008). A major reason why homology is such a difficult concept in eye evolution is that developmental genetic networks are likely to have been co-opted by cells at new locations (Nilsson 2004; Vopalensky & Kozmik 2009). True homology requires that a feature, shared by different species, has an unbroken history back to the same feature in a common ancestor. This implies that the homologous structure must develop from cells in the same ontogenetic cell linage or from corresponding lineages in other body segments (serial homology). Structures that develop from cells with different cell division pedigrees are not homologous, but may very well be co-opted by homologous control genes (True & Carroll 2002; McLennan 2008). Ectopic eyes have been experimentally induced in various positions by mis-expression of developmental genes in both *Drosophila* and *Xenopus* (Halder *et al*. 1995; Onuma *et al*. 2002). Ectopic eyes are artificially induced cases of co-option, and the number of different developmental genes that are capable of inducing ectopic eyes offers an indication that co-options are frequent in evolution.

Co-option in nearby cells, with similar but not identical pedigrees, is likely to be more easily induced, and such co-option would also be particularly difficult to distinguish from homology. In addition, cell-type-specific gene expression will be deceptive in cases in which whole sets of genes are co-opted as assemblies, and what appear to be many independent similarities are in fact only a single similarity with a certain likelihood of falsely indicating homology. The problems are further compounded by the modular nature of developmental genetic networks (Oakley 2003). Co-option of components such as opsins, G-proteins and ion channels from different systems can generate new systems with a bricolage of developmental gene homologies. Eyes on the mantle edge of clams and on the branchial tentacles of fan worms are likely to be the result of extensive co-option and genetic bricolage (Nilsson 1994; Oakley 2003). Co-option and bricolage should not be understood as exceptions, but rather as standard evolutionary mechanisms that contribute greatly to evolvability (Lenski *et al*. 2006).

On the basis of existing morphological and molecular evidence, together with the discussion on evolutionary processes mentioned earlier, it is possible to sum up some conclusions on the major branches of eye evolution. It seems clear that opsin-based light sensitivity has a single origin and was duplicated into multiple systems very early in animal evolution (Plachetzki *et al*. 2007). The paired cephalic eyes of invertebrates show many signs of homology, such as r-opsins, a G_q_ transduction cascade and receptors with rhabdomeric morphology (Arendt 2003, 2008). The separate larval and adult cephalic eyes of many invertebrate phyla (Arendt & Wittbrodt 2001) may be regarded as a case of sequential homology, but which of these is the original eye remains an open question awaiting better knowledge about the life histories of the common animal ancestors. Vertebrate eyes are probably best understood as a bricolage of components (Arendt *et al*. 2004; Arendt 2008), possibly because early vertebrates went through a phase in which ancestral systems were simplified before they again became complex (see also Lamb *et al*. 2007). Non-cephalic eyes, in general, are likely to result from co-option of control genes or recruitment and elaboration of non-visual photoreceptive systems. Cnidarian eyes show links to different photoreceptor classes in bilaterians, but it is yet too early to be confident about the level of homology between eyes in these two major animal branches. Next, I investigate whether the selective advantages that guided early eye evolution may help lift the fog from some of the issues discussed earlier.

## 2. Early sensory tasks and their functional requirements

The widespread occurrence of eyes or ocelli suggests two related conclusions: first, that eyes or ocelli offer a significant functional advantage for the vast majority of animal lifestyles, and second, that eyes or ocelli appeared very early in animal evolution. Is it one common role or are there many different roles? To answer this question, it is necessary to define the types of information that light can potentially provide. The general ambient light intensity varies with the time of day, time of year, lunar cycle, weather, water depth and position in relation to shading structures. It is reasonable to assume that the evolution of sensory tasks based on light sensitivity started as non-directional monitoring of the ambient luminance and that this formed the basis from which directional sensitivity and true vision evolved. Non-directional monitoring of the light intensity can be used as entrainment for a circadian clock; it can serve photokinesis tasks such as a shadow response or a depth control in water, or it can act as a surface indicator for a burrowing lifestyle. As soon as a receptor cell gains some directionality, it can control direct phototaxis and operate as an optical statocyst for controlling body orientation. Multiple photoreceptor cells, pointing in different directions, are the basis for true vision, and this allows the vast numbers of visual tasks performed by arthropods, cephalopods and vertebrates. A summary of the more primitive roles taken by animal light receptors is given in table 2. For a listing of more advanced visual tasks, see Land & Nilsson (2006).

**Table 2.** Properties of photoreceptor systems and possible sensory tasks. Shadow detection is not included because although it does not require directionality, it does require fast response and adaptation.

properties of sensory system	sensory tasks
non-directional light sensitivity: no screening pigment, slow response, large dynamic range, no adaptation	circadian entrainment
	depth gauge
	UV warning
	surface detection: burrowing lifestyle
directional light sensitivity: screening pigment, moderate response speed, limited dynamic range, adaptation	phototaxis
	optical statocyst
spatial vision: multiple receptors, pigment cup, low resolution, moderate response speed, limited dynamic range, adaptation	navigation in relation to inanimate structures
spatial vision: multiple receptors, focusing optics, high resolution, fast response, limited dynamic range, adaptation	interaction with other animals

For each of the primitive roles of animal light sensitivity, there are functional requirements in terms of absolute sensitivity, spectral sensitivity, speed and intensity adaptation. These requirements are of central importance here because they must have generated the selection driving evolution of cell structures and molecular mechanisms in photoreceptor cells and supporting cells. Analyses of these requirements are given below, starting with the basic requirements for light sensitivity and working through sensory tasks based on ambient light levels, via directional light reception, and finally to true (spatial) vision.

### (a) Light sensitivity

Detection of light by animal opsins implies that 11-*cis* chromophore is consumed. To maintain sensitivity, active chromophores must be replenished at a rate comparable to consumption. Direct sunlight corresponds to 10 million photons of visible light per second reaching each square micrometre of the earth's surface, and this consumes the 11-*cis* chromophore at an extreme rate in exposed photoreceptor cells. However, spontaneous (thermal) regeneration from an all-*trans* to an 11-*cis* retinal only occurs at very low rate (Deng *et al*. 1991). Using light also to flip the chromophore back from all-*trans* to 11-*cis* configuration solves the problem, but for free chromophore, this requires ultraviolet (UV) radiation that is absent even at moderate depths in the sea. The photoisomerases, which are non-sensory opsins, have the property of regenerating the chromophore in the presence of visible light, and as such, they are complementary to c-opsins, which lack this ability. The r-opsins are different in that they act both as receptor proteins and photoisomerases, such that the chromophore can remain bound to the opsin at all times. If it is assumed that the complex photopigment cycle of vertebrates, with enzymatic dark regeneration (Lamb & Pugh 2004; Kusakabe *et al.* 2009), is a later acquisition, then the functional evolution of visual pigments can be tentatively reconstructed.

**Figure 3.**
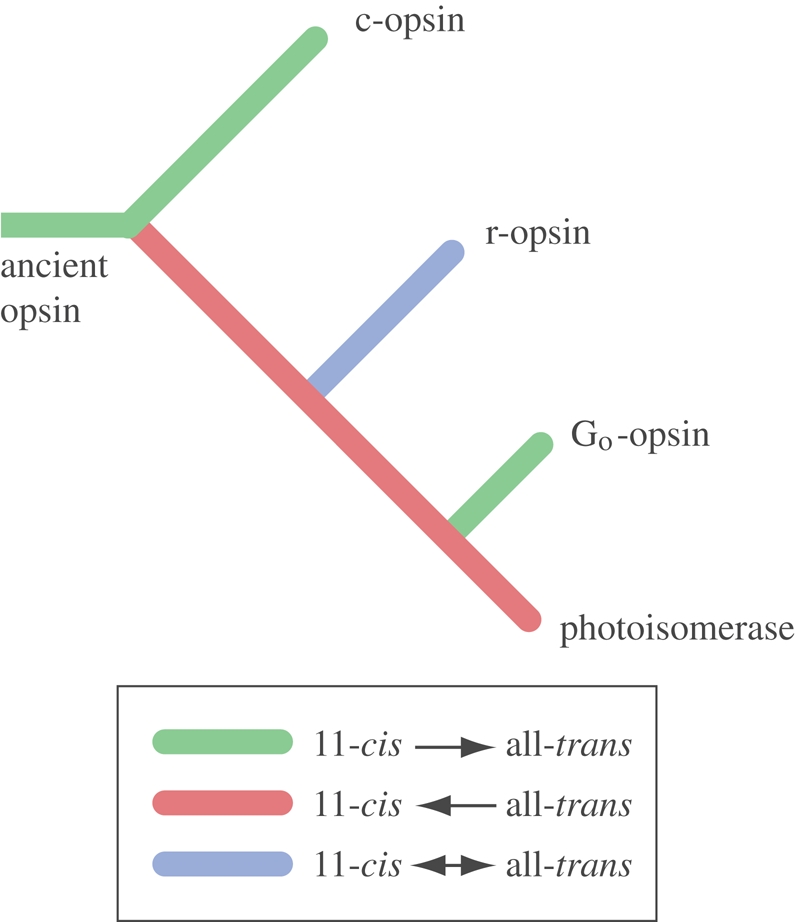
Hypothetical evolution of opsin function. For the underlying arguments, see text. The phylogenetic tree is according to Plachetzki *et al*. (2007).

The ancient opsin, from which c-opsins, r-opsins and photoisomerases/G_o_-opsins evolved, may have been a pure receptor protein (like a c-opsin), a pure photoisomerase or a protein with combined properties (like an r-opsin). Of these three possibilities, the ancestral opsin is unlikely to have been a photoisomerase because it is hard to see a need for such an enzyme if there is no receptor protein producing its substrate. An origin as a combined (bi-stable) protein would lead to two alternative scenarios, where either a photoisomerase or a pure receptor protein could evolve from one of the copies after a gene duplication. The latter option is unlikely because a pure receptor protein (c-opsin type) would be inferior to the original bi-stable protein before a photoisomerase evolved. If, on the other hand, a photoisomerase evolved from a gene copy of an original bi-stable pigment, then the c-opsin class would evolve last and form a clade with either r-opsins or G_o_-opsins/photoisomerases, and neither of these phylogenies are supported (Plachetzki *et al*. 2007; figure 3). The only viable option is thus that the ancestral opsin was a pure receptor molecule similar to present day c-opsins. If a bi-stable pigment came second, it would likely lead to the loss of the original gene because a pure receptor protein, without the complement of a photoisomerase, would be vastly inferior to a bi-stable opsin. The only remaining scenario is that an ancestral opsin had c-opsin properties and that the first gene duplication led to the evolution of a photoisomerase that would form an efficient functional pair together with the ancestral opsin. The r-opsin class would then have evolved after duplication and modification of the photoisomerase, and a similar event would have happened again to generate the G_o_-opsins. Such a scenario is consistent both with the opsin phylogeny (Plachetzki *et al*. 2007) and with functional arguments (figure 3).

The original split to generate a photoisomerase together with the receptor protein would have happened very early, before the split of cnidarians and bilaterians. If present day cnidarians use c-opsins in their eyes, as suggested by Kozmik *et al*. (2008), the cnidops clade would be expected to contain photoisomerases that work together with the c-opsins. The observation that jellyfish opsins display bleaching (O'Connor *et al*. 2009) leads to the hypothesis that cnidarian photoreception is based on separate receptor and enzyme proteins.

A related question is why opsins have become the only type of visual photopigment, whereas animals use both opsins and cryptochromes for non-visual photoreception. This is remarkable because cryptochromes were inherited from the unicellular predecessors of animals (Rubin *et al*. 2006), but type II opsins are unique to animals (Larusso *et al*. 2008) and must thus have evolved in an ancestor that already possessed cryptochromes. Spontaneous regeneration of cryptochromes occurs at a reasonable rate (Kennis *et al*. 2004; Bouly *et al*. 2007; Losi 2007; Kao *et al*. 2008), and in this sense, cryptochromes would have been superior to the ancient bleachable opsin. There are thus likely to be other reasons that made opsins a viable alternative to cryptochromes. It is possible that speed and amplification in the signalling pathway gave opsins an edge for visual systems in which speed and sensitivity are more important than they are for non-visual roles. An alternative explanation is that the spectral sensitivity of opsins is more suited to vision in the green light that best penetrates coastal water (500–550 nm), whereas the blue and UV sensitivity of cryptochromes (less than 500 nm) is adequate for non-visual tasks (Gehring & Rosbash 2003).

### (b) Ambient light tasks

**Figure 4.**
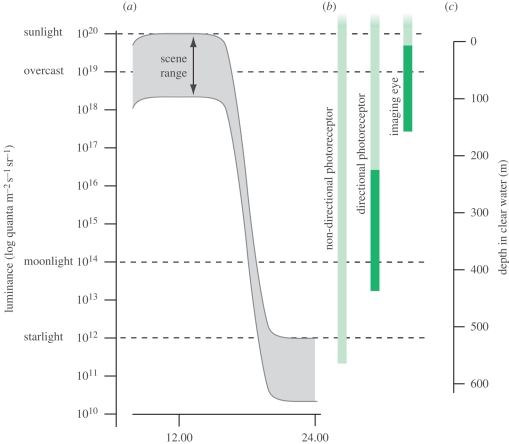
Natural luminances and the sensitivity of eyes. (*a*) Part of the diel light cycle, plotted on a log intensity scale. The range of luminances within a scene is constant, but slides up and down the log scale between day and night. Under overcast conditions, the whole luminance function is shifted down by up to 1 log unit. (*b*) The operational range of photoreceptors was modelled for non-directional monitoring of ambient luminance, directional scanning phototaxis and spatial vision (for calculations and data, see electronic supplementary material). *Light green* indicates the operational range for a photoreceptor without membrane extensions, and *dark green* is for the corresponding photoreceptor with a realistic amount of membrane stacking. The non-directional photoreceptor works to below starlight levels without any membrane stacking, but imaging eyes cannot discriminate luminances within a scene even in bright sunlight unless the photoreceptor membrane is extensively stacked. By combining large lenses, moderate resolution and slow vision, nocturnal and deep-sea animals can use their eyes at lower luminances than indicated by the rightmost green bar (Warrant 1999). (*c*) The depth in clear ocean water is plotted on the same luminance scale, assuming sunlight at the surface.

The luminance generated by natural light sources varies over an impressive 8 log units between sunlight and starlight (Land & Nilsson 2002). A heavily overcast sky will reduce the luminance by about a log unit, making approximately 9 log units change in average scene luminance from a sunny day to an overcast moonless night (figure 4). The difference in luminance between different parts of a scene is much less, typically 1.5–2 log units (the light source not included). Depth in water has a dramatic effect on light intensity. Even the clearest ocean water attenuates light by approximately 1.5 log units for every 100 m, and in coastal water, attenuation is many times higher.

The 24 h light cycle has obvious relevance to most animals. Even the first metazoans would have benefited from knowing when their food organisms were engaged in photosynthesis. Present day planktonic organisms, both animals and their food organisms, are involved in diurnal vertical migrations to find optimal conditions for feeding/photosynthesis, avoiding being eaten, access to nutrients, dispersal and a number of other factors (Harris 1953; Putzeys & Hernández-León 2005). Many of these factors would have motivated vertical migrations also at the time of the first metazoans, before visually guided predation and macroscopic animals evolved. Non-directional monitoring of light intensity is sufficient for controlling vertical position in aquatic organisms, but it requires a combination of photokinesis and an orientation to gravity (passive or through geotaxis; Häder *et al*. 2005; Roberts 2006; Richter *et al*. 2007). Light is an equally important cue for epibenthic or partly burrowing animals, and light-entrained circadian clocks and optical depth gauges would have offered selective advantages to urmetazoan or urbilaterian ancestors irrespective of whether they were swimming or crawling.

Using light as a measure of depth in water is complicated by the fact that the luminance is determined both by the time of day and by the depth. Unless an organism strictly follows isolumes, it is necessary to separate the causes of any luminance changes, such that time of day and depth in the water can be disentangled. There are two obvious ways for vertically migrating plankton to solve the luminance ambiguity. First, a circadian rhythm can anticipate the time of day, and setting of the clock can be done over many light cycles, such that the impact of depth variations can be minimized. The other possibility is to use the fact that the spectral composition of light varies in different ways depending on time and depth (Lythgoe 1979). The latter has a much stronger influence on spectral composition: clear oceanic water becomes gradually bluer with depth and coastal water becomes greener. Two spectrally different photopigments can form a very robust depth gauge, which is largely independent of the diel light cycle. Another reason for evolution of more than a single photopigment is that high sensitivity requires a spectral peak close to the maximum transmission of water, whereas the detection of UV light might be useful for avoiding radiation damage of proteins and genetic material that may occur close to the surface during the day.

There would thus have been good reasons for the early metazoans to acquire a light-entrained circadian clock and spectrally different photopigments to measure water depth and warn against UV exposure. In all these tasks, it is the absolute intensities that carry the information. This means that the receptors must cover a very large dynamic range and that adaptation is not desirable. The intensity changes are also slow, which calls for slow responses integrating over considerable time. These are typical properties of non-visual photoreceptors (Gotow & Nishi 2008). For UV warning, photoreceptors could have a smaller dynamic range and faster response, but adaptation should also be absent for this task.

A depth gauge based on comparison between the signals from two spectrally different opsins might have been an early reason, not just for spectrally diverse opsins, but also for chromatic antagonism and different transduction cascades. Receptors in the parietal eye of lizards, although not used as a depth gauge, possess a mechanism for chromatic antagonism in a single cell (Solessio & Engbretson 1993), and the two opsins act antagonistically through different transduction cascades with opposite effects on the ion channels (Su *et al*. 2006). In early metazoans, the evolution of similar chromatic mechanisms might have been driving divergence in both opsins and transduction mechanisms and later been segregated into different cell types.

### (c) Directionality tasks

The direction of light contains an enormous amount of information about the surrounding world. A first evolutionary step to begin exploring this information is to obtain some form of partial shielding of the photoreceptor cells. Directional light sensitivity may have started as a by-product of protective body pigmentation. Even though screening pigment is a typical part of eyes and ocelli, the need for pigmentation did not arise with the evolution of vision. Pigments occur in all organisms that are exposed to light, and they shield the cells from damage by short-wavelength light. The reason why biological pigment molecules, such as melanins and pterins, can absorb light without being damaged themselves is that the highly conjugated double bonds allow energy to be dispersed over the molecule and easily dissipated as heat. For the very same reason, biological pigments remove harmful free radicals and protect the cell from oxidative damage (McGraw 2005). Short-wavelength light is one cause for free radicals, and biological pigments thus have a dual role in protecting against the adverse effects of light. This means that screening pigments must have been common cellular constituents long before vision evolved in animals.

If body pigmentation provides some rudimentary directionality to a photoreceptor cell, it is easy to see how selection could pick up on it and improve the directionality by synthesis of screening pigment in the receptor cell or in adjacent cells. As soon as there is some directionality (figure 1*a*–*c*), the animal's own movements will allow for comparisons of the light intensity in different directions. This information can be used for phototactic orientation towards or away from light (for a discussion of phototaxis in planktonic organisms, see Jékely 2009), or it can function as an optical statocyst for controlling body posture in relation to the general direction of light. Changing from non-visual tasks to phototaxis requires major changes in the properties of the receptor cells. The relevant information is no longer the absolute intensities, changing by 8 log units between day and night, but rapid changes (seconds), covering 1.5–2 log units superimposed on the 24 h light-cycle. To extract information for phototaxis, the receptor cells need a fast response, corresponding to the animal's pattern of movement, and a high contrast sensitivity to detect the comparatively small luminance differences in different directions. A mechanism for adaptation, to remove the 24 h light cycle from the response, is also necessary.

These properties require both quantitative and qualitative modifications of the detection and signalling mechanisms in the receptor cells; but more importantly, the reduced angle through which light is received, together with the much shorter integration time, results in a dramatic reduction in the number of photons detected during each integration time. The need to discriminate smaller differences in intensity simultaneously calls for larger photon samples (Land & Nilsson 2002). To assess the ability to discriminate between relevant intensity differences, I calculated the required minimum intensity to perform specific tasks such as detection of the diel intensity rhythm, simple phototaxis or true spatial (image) vision (for details, see electronic supplementary material). The calculations were based on five realistic models: one of a non-visual photoreceptor, two of different directional ocelli used for phototaxis and two of spatially resolving eyes (a lens-less cup eye and a small camera-type eye). A central question motivating the modelling was whether membrane stacking, such as microvilli, lamellae or discs, is necessary for the different tasks.

The results were unexpected (figure 4; see electronic supplementary material). Non-directional photoreception for detecting the diel light cycle can accommodate the full, 8 log unit range of luminance without any morphological membrane specializations. Even if the photopigment density is several log units lower than in vertebrate or insect visual cells, the sensitivity would be high enough to detect moonlight. For non-visual luminance monitoring, there is thus no need for cilia or microvilli. Indeed, non-visual photoreceptors often lack these conspicuous structures altogether (Gooley *et al*. 2003; Gotow & Nishi 2007, 2008; Van Gelder 2008). Modelling also demonstrates that phototaxis, which requires directionality with wide angular sensitivities and intermediate integration times, can function without membrane stacking, but only during the day, and in rather shallow water (figure 4; see table S1, electronic supplementary material). The fact that acoel flatworms have ocelli without cilia or rhabdomeres supports the conclusion that directional photoreception (presumably for phototaxis) does not require membrane stacking (Yamasu 1991). For use at low crepuscular intensities in deeper water or in very turbid water, moderate stacking of membrane will help extend the range of intensities where phototaxis can be used (figure 4).

**Figure 5.**
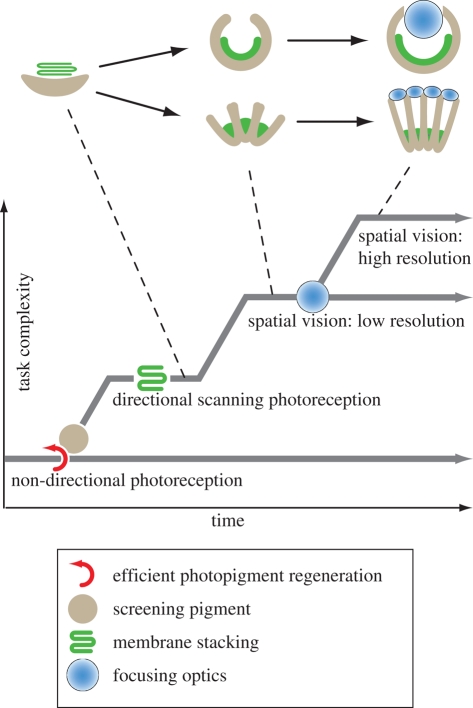
Key innovations in eye evolution. Directional photoreception is assumed to have evolved from non-directional monitoring of ambient luminance by a cell duplication event and an opsin gene duplication leading to a receptor opsin and photoisomerase pair of proteins for efficient chromophore regeneration. This was followed by the introduction of screening pigment and soon also by membrane stacking to allow for better contrast discrimination, increased speed and more directional photoreception. Multiple receptor cells would then allow for true spatial vision and the scanning mode of operation could be abandoned. The single-chambered and compound eyes would have to evolve independently from directional ocelli. To collect enough photons for spatial vision with higher resolution, lenses must be introduced, but the new high-resolution tasks are expected to add to rather than replace the older low-resolution tasks.

Directionality, fast response and adaptation acting as a temporal high-pass filter are important properties for a photoreceptor that serves phototaxis or functions as an optical statocyst (figure 5). These properties pass information about the angular distribution of light in the environment and remove information about the general ambient luminance. A photoreceptor cell that develops a role for directional light measurements must, therefore, abandon any original task for monitoring the general ambient luminance. But because entrainment of biological clocks is likely to remain important, directional photoreception would probably only evolve after a non-directional photoreceptor has been duplicated. The evolution of phototaxis and optical statocysts are thus likely to have served as early reasons for multiple photoreceptor systems in early metazoans.

### (d) Imaging tasks

A single directional photoreceptor relies on body movement to acquire information about the angular (spatial) distribution of light, and this interferes with the ability to detect changes in the environment. With different photoreceptors pointing in different directions, spatial information can be collected simultaneously without body movement. With just two receptors pointing in different directions, there is in principle an image, and by adding more receptors, the eye can increase the amount of information virtually without limit. Spatial vision is also the most information rich of all senses, and it offers sensory guidance to the most sophisticated of animal behaviours. There can be no doubt that the step from a directional photoreceptor to the first pit or cup eye was of pivotal importance for animal evolution.

Evolving a spatially resolving eye from a directional photoreceptor means that the angle seen by each receptor will have to shrink, and this dramatically reduces the rate at which photons are detected. Going from the 180° fields of the larval eye of *Platynereis dumerilii* (figure 1*b*; Jékely *et al*. 2008) to a 25° degree field in a small pigment-cup eye (figure 1*d*) involves a 42 times reduction in photon detection rate. This loss in sensitivity is a major obstacle in the evolution of spatial vision. The problem is compounded by the fact that the integration time will have to be reduced along with the receptor's field of view in order to keep motion blur at tolerable levels. In addition, the contrasts of interest are smaller for spatial vision than for phototaxis, which calls for larger photon samples per integration time (see electronic supplementary material for a discussion of photon sample size). A consequence of this rapidly increasing need for photons is that stacking of the photoreceptor membrane becomes an absolute prerequisite for the evolution of spatial vision. Even daylight would be too dim for a pigment-cup eye without any stacking of the photoreceptive membrane (figure 4; table S1, electronic supplementary material). Rhabdoms and ciliary specializations must thus have been in place, at least to some degree, before the first spatially resolving eyes evolved.

Because each double membrane layer can absorb not more than 0.1 per cent of the light (see electronic supplementary material), there is a great potential gain in folding and stacking the membrane. But when the membrane layers become numerous, the gain produced by each new layer declines. A human rod has some 1300 discs, and a blowfly rhabdom has about twice that number of microvillar layers. Compared with an unfolded cell membrane, this stacking involves a sensitivity gain of 2–3 log units (see equations in electronic supplementary material). If such a sensitivity gain were spent on compensating for the sensitivity loss caused by decreasing angular sensitivities, how much spatial resolution would it buy? The answer is somewhat disappointing. Starting from the 180° receptive field of a directional phototaxis system, the 2–3 log units of extra sensitivity, obtained by membrane stacking, is sufficient only to take the angle down to approximately 10°, and if shorter integration times and larger photon samples are taken into account, the resolution improves even less. Angular sensitivities of 10–20° are sufficiently small for navigation in relation to inanimate structures of the surrounding world, but they need to come down significantly to allow for more advanced visual tasks such as pursuit of prey, predator detection or mate recognition.

After exhausting the benefits brought about by membrane stacking, another strategy is obviously necessary for continued refinement of visual resolution. That strategy is the introduction of focusing optics. By introducing a lens, the area detecting light can be shifted from the area of the receptor cell to the area of a lens (figure 4; table S1, electronic supplementary material). Because animal cells are only 5–10 µm across, and animal lenses can have diameters in the range of millimetres or even centimetres, the detection area can be increased by up to 8 log units. This allows for a huge boost in photon catch, which is needed for the high resolution of arthropod, cephalopod and vertebrate eyes, and it provides enough sensitivity to tune eye design to nocturnal or deep-sea use. For a discussion of more advanced visual tasks, see Land & Nilsson (2006).

Lenses can be introduced gradually if there is tissue filling the cavity of a pigment-cup eye (figure 1*e*). By expression of suitable proteins, the refractive index can be increased and distributed such that a high quality lens is formed (Nilsson & Pelger 1994). Recent work on the eyes of box jellyfish (O'Connor *et al*. 2009) suggests that the cells filling the cavity of vesicular eyes may have multiple functions and that focusing is not the primary function. Before focusing properties evolve, the obvious way to form an image is by shading in a pigment pit or cup. A simple way of forming the pigment cup is to make the receptor cells line the exterior of a growing lump of transparent cells. Such a mechanical function would require some rigidity of the transparent cells, and this would naturally pre-adapt the cup eye for transition to a lens eye. Another and possibly even older function of vitreous cells in the cavity of pit or cup eyes is to serve as UV filters for protecting against radiation damage to the receptor cells. It thus seems that lens evolution does not require any unusual circumstances, and the reason why many invertebrates have a ‘füllmasse’ rather than a lens in their eyes is likely to be that selection has not favoured any visually guided behaviours that require high resolution in these species.

Spatial vision can originate not only by multiplying receptor cells inside a pigment pit, but an equally viable option is to multiply the entire structure including the pigment pit. In flatworms, these two possibilities are both represented (Kuchiiwa *et al*. 1991). At early stages of eye evolution, it is not easy to see that either of the two alternatives would be much better than the other. Later, however, when lenses have been introduced, the single chambered solution turns out to outperform the compound eye by a rather wide margin (Kirschfeld 1974). One reason for the difference is that, in eyes of comparable size, the many lenses of a compound eye must be much smaller than the single lens in a camera type eye.

The approximately 1.7 log unit luminance range within natural scenes will slide up and down the intensity scale between day and night, when clouds and weather change, and when an animal changes its depth in the water or moves between shaded and exposed areas. To detect the smallest possible contrast within the luminance range of the scene, it is necessary for imaging eyes to adapt, such that the response/intensity function matches the luminance range at all times (Laughlin 1981). This means that information about the absolute intensity is removed from the visual system. Yet, some mechanisms of adaptation, such as the pupil diameter and the amount of neural summation (Warrant 1999), should ideally be controlled by the absolute intensity. As a consequence, non-visual photoreception serves a function in imaging eyes, and it is no surprise that visual interneurons, such as some ganglion cells in vertebrate eyes, are intrinsically light sensitive, without directly contributing to vision (Gooley *et al*. 2003). These cells display similarities to other non-visual photoreceptors in the slow time course of the response (Do *et al*. 2009), but it appears that they do not entirely lack adaptation (Wong *et al*. 2005). For an evolutionary understanding of intrinsically light-sensitive interneurons in the visual pathway, it is important to note that this non-visual function is likely to represent an elaboration of the visual pathway, and, as such, it is not necessarily a conserved function dating back to pre-visual ancestors.

## 3. Synthesis and outlook

The driving force behind sensory evolution is the addition of new sensory tasks that provide animals with new responses to external information. This reasoning leads to an understanding of sensory evolution as a task-punctuated process. For the evolution of each new task, the sensory system will be subject to selection that works towards extraction of a specific subset of sensory information. New sensory tasks evolve by modification or elaboration of systems that serve already existing sensory tasks. In some cases, the new task serves a similar purpose as an older task, leading to the replacement and loss of the older task. An example of replacement is the transition from directional photoreceptors used for phototaxis to spatial vision with multiple photoreceptors. Here, it is likely that the ancestral scanning mode of data acquisition was simply replaced by the simultaneous acquisition of spatial information. In other cases, a new task was introduced without making the previous task obsolete. The evolution of directional photoreception for phototaxis from non-directional monitoring of the diel light cycle is an obvious example in which the new sensory task does not infringe on the value of the older task. In such cases, a duplication event must have initiated the evolution of the new task.

On the assumption that evolution, in general, would proceed from tasks with small demands on molecular machinery and morphological structures to tasks with gradually more extensive requirements, the evolutionary sequence of early tasks leading to true vision can be reconstructed with some confidence. This sequence starts with non-visual photoreception for circadian control or water-depth control, followed by directional photoreception for phototaxis or body orientation, which then becomes replaced by true spatial vision. In terms of structures, this would have corresponded to a sequence from photoreceptor cells without membrane specializations, via directional shading by screening pigment structures, either in the photoreceptor itself or in an associated cell, through to the development of membrane folding, which would open up for enough sensitivity to evolve the first true eyes with spatial vision (figure 5).

The duplication events that led to the different opsin classes are likely to correspond to the introduction of new or modified sensory tasks. As argued in this paper, the original split between c-opsins and all other opsins may have generated a photoisomerase enzyme to improve the efficiency of the original opsin. If this was the case, the gene duplication may have led to improved function of an existing sensory task rather than the duplication into a new task and an old task. Because this opsin duplication predates the split between Cnidaria and Bilateria (Plachetzki *et al*. 2007), the sensory task requiring more efficient regeneration would have existed in the last common ancestor of the two animal groups. It is hard to see that rapid regeneration would be a crucial property of non-visual luminance monitoring, especially since adequately efficient cryptochrome-based systems probably were already in place (Rubin *et al*. 2006). A distinct possibility is that the rapid regeneration of photopigment would have facilitated the evolution of directional photoreception.

The evolution of membrane stacking, be it in the form of cilia, microvilli or diverticula, is expected to have been a prerequisite for the transition from directional photoreception to the first real eyes. As a consequence, if stacking based on microvilli and cilia could be demonstrated to be of independent origin, it would imply that spatial vision originated more than once. Unfortunately, there has not been much development on this question since the intense debate of three decades ago (Salvini-Plawen 1982; Vanfleteren 1982). Even though the majority of photoreceptor cell types that have been investigated can be neatly classified as rhabdomeric or ciliary, there are still a number of intermediate cases and cells that change from one type to the other during ontogeny (see §1*b*). There is an obvious risk that the type of membrane stacking has been more evolutionarily plastic than assumed and that the distinction between rhabdomeric and ciliary receptors has been given too much significance. Hopefully, a broad comparative identification of the opsin type in photoreceptor cells will resolve this issue.

Jékely *et al*. (2008) suggest that the rhabdomeric ocelli involved in phototaxis in the planktonic larvae of polychaetes (figure 1*b*) represent the ancestral bilaterian condition. This is an attractive idea, but it does not bring any clarity into why polychaetes have ciliary photoreceptors in the brain involved in circadian control (Arendt *et al*. 2004). The ciliary membrane stacking of these non-visual photoreceptors strongly suggests an evolutionary history as directional photoreceptors (see §2*c*, figure 4; see electronic supplementary material). This points to a much more ancient role for directional photoreception or indicates that it evolved independently in two different systems and that the directionality (screening pigment) was secondarily lost when one of the systems regained its original role in luminance monitoring. Yet another possibility is that the association between photoreception and cilia did not originally arise as a means of membrane stacking, but that it is founded in an ancient functional connection between sensory control of ciliary beating. The self-contained sensory-motor function of the ocellar cells in box jellyfish larvae (figure 1*a*; Nordström *et al*. 2003) would then be a better representative of the first directional photoreceptors in animals.

The many cases of rhabdomeric and ciliary membrane stacking in cells that are not associated with screening pigment structures (e.g. Purschke *et al*. 2006) support the idea that non-directional photoreceptive systems have evolved repeatedly from ancient directional systems, presumably by duplication and subsequent loss of the screening pigment in one of the copies. The picture is further complicated by the simple ocelli in acoel flatworms (figure 1*c*), which lack membrane stacking, but are associated with a pigment cell (Yamasu 1991). Acoel flatworms are believed to be basal bilaterians, and they have a direct development without planktonic larvae (Ruiz-Trillo *et al*. 1999, 2002; Telford *et al*. 2003; Philippe *et al*. 2007). It is tempting to suggest that this would date back to a system for phototaxis in benthic crawling Urbilateria and not in planktonic ancestors as proposed by Jékely *et al*. (2008).

From the current knowledge, it seems that it is possible to generate an army of different hypothetical evolutionary scenarios, none of which fully or easily accounts for the different sets of photoreceptors in different groups of animals. The problem, as it stands, is that the morphological evidence displays a semi-consistent pattern with numerous exceptions and cases that are hard to classify and that the molecular/genetic evidence on opsin types and transduction cascades is yet available only from a limited set of animal groups and species. There is an obvious need for more comparative knowledge on opsin types, transduction cascades and other molecular cues before the morphological diversity can be understood. A functional context, and the concept of task-punctuated evolution introduced here, is necessary not only to guide the formation of hypotheses, but also to understand rather than just describe the evolution of light sensitivity and vision.
